# Supplementary material for: Is all greenspace created equal? Assessing the relationship of public parks and greenness on academic achievement in Washington state
Source: PLoS One. 2026 May 6;21(5):e0347301. doi: 10.1371/journal.pone.0347301 (PMC13148709; doi:10.1371/journal.pone.0347301)
Supplement: S1 Table — (PDF) [file pone.0347301.s001.pdf]

Supporting information

S1 Table. Regression results by grade-level for 800 m buffer area and 800 m spring NDVI

Grade Level	3		4		5		6		7		8		10		11	
**Outcome variable: Percent met standard**	**Coef.**	**SE**	**Coef.**	**SE**	**Coef.**	**SE**	**Coef.**	**SE**	**Coef.**	**SE**	**Coef.**	**SE**	**Coef.**	**SE**	**Coef.**	**SE**
Park within 800 m	0.81	0.75	0.24	0.67	0.13	0.68	1.08	1.13	3.23*	1.67	5.49***	1.68	1.08	1.94	.6	2.36
NDVI 800m Spring	5.61	11.13	12.96	9.51	10.1	11.0	5.89	8.93	16	11.66	17.92	10.99	6.11	18.18	-.43	24.84
Percent Low Income students	-.39***	0.4	-.38***	0.4	-.33***	0.3	-.31***	0.05	-.36***	0.04	-.35***	0.04	-.46***	0.07	-.28***	0.08
Percent female students	.06**	0.04	.018	0.4	-0.01	0.05	.03	0.06	.04	0.1	.09	0.08	-.11	0.08	.04	0.13
Percent White	.001	0.05	-.05	0.04	-.01*	0.05	-.01	0.2	.12	0.08	.03	0.1	-.2	0.15	-.01	0.2
Percent Native Indian	-0.06	0.11	-.3***	0.11	-.31*	0.17	-.97***	0.19	-.31	0.27	-.55**	0.23	-.34*	0.16	-.08	0.21
Percent Asian	.16**	0.07	.17***	0.05	.12*	0.07	0.33***	0.09	.37***	0.09	.32***	0.11	.22*	0.11	.45**	0.19
Percent African American	-.03	0.06	-.19***	0.07	-.34***	0.09	-0.3**	0.12	-0.04	0.11	-.19	0.16	-.35	0.22	-0.04	0.21
Percent Hispanic	-.12*	0.07	-.15*	0.08	-.24***	0.08	-0.16	0.1	.08	0.11	-.03	0.13	-.48***	0.14	-.02	0.21
Percent Hawaiian	-.08	0.17	-0.09	0.19	-.38***	0.14	-0.37	0.23	-.45	0.36	-.58**	0.28	-.56**	0.26	-.18	0.30
Teacher average years of experience	.83***	0.28	.79***	0.22	.77***	0.23	0.49*	0.25	.31	0.24	-.02	0.34	-.04	0.37	-.05	0.32
Percent teachers with Master's degree	-.09*	0.05	-.06*	0.04	-.04	0.04	0.13**	0.06	.15***	0.03	.15***	0.05	.26***	0.08	2**	0.08
Log general per pupil funding	-.26**	0.11	-.37***	0.09	0.02	0.1	0.16	0.11	.76***	0.13	.45***	0.16	.16*	0.38	-2.17***	0.42
Log school district property tax	-4.35	4.09	-8.36**	3.83	-4.4	3.11	0.28	7.16	2.16	5.6	8.7	7.33	4.38	7.19	8.26	12.93

Rural Dummy	0.1	1.98	0.02	2.32	-0.78	1.88	2.91***	0.76	-0.24	1.5	-2.88	1.83	4.27	4.36	7.6***	2.76
Total students (school)	0.01**	0.005	.009*	0.005	.01*	0.005	0.01	0.004	0.01**	0.004	.01***	1.83	.01***	0.002	0.002	0.002
Adjusted R-squared	0.6		0.63		0.59		0.62		0.62		0.56		0.59		0.24	
Observations	5,603		5,600		8,400		2,962		2,013		2,999		1,126		1,308	

Note: Significance: ***p < 0.01, **p < 0.05, and *p < 0.1. The results reported here are based on all grades (except grades 1, 2, 9, and 12) within each school level that took the math, science, and/or language arts assessment tests across all five years of analysis. Results from the English language assessment test are excluded from the regression results. All models include district, county, and year fixed effects.
