# Supplementary material for: Is all greenspace created equal? Assessing the relationship of public parks and greenness on academic achievement in Washington state
Source: PLoS One. 2026 May 6;21(5):e0347301. doi: 10.1371/journal.pone.0347301 (PMC13148709; doi:10.1371/journal.pone.0347301)
Supplement: S2 Table — (PDF) [file pone.0347301.s002.pdf]

Supporting information

S2 Table. Comparison across 400 and 250m buffer Spring & 800m, 400m, and 250m Fall NDVI with 800m buffer measure of greenspace for middle school

Outcome variable: Percent met standard	NDVI 400m Spring		NDVI 250m Spring		NDVI 800m Fall		NDVI 400m Fall		NDVI 250m Fall	
	**Coef.**	**SE**	**Coef.**	**SE**	**Coef.**	**SE**	**Coef.**	**SE**	**Coef.**	**SE**
Park 800m Dummy	4.02*	1.58	4.03**	1.58	4.05**	1.61	4.03**	1.59	4.03**	1.58
NDVI (400m Spring)	3.99	11.21	-		-		-		-	
NDVI (250m Spring)	-		0.13	10.56	-		-		-	
NDVI (800m Fall)	-		-		8.63	10.7	-		-	
NDVI (400m Fall)	-		-		-		-1.36	9.11	-	
NDVI (250m Fall)	-		-		-		-		-4.66	8.94
Percent Low Income students	-.35***	0.03	-.35***	0.03	-.35***	0.03	-.35***	0.03	-.35***	0.03
Percent female students	.09	0.07	.09	0.07	.09	0.07	.09	0.07	.09	0.07
Percent white	.07	0.07	.07	0.08	.07	0.07	.07	0.07	.07	0.07
Percent Native Indian	-.51**	0.22	-.52**	0.22	-.5**	0.23	-.52**	0.23	-.53**	0.23
Percent Asian	.35***	0.09	.34***	0.09	.35***	0.09	.34***	0.09	.34***	0.09
Percent African American	-.14	0.12	-.14	0.12	-.14	0.12	-.14	0.12	-.15	0.12
Percent Hispanic	-.01	0.12	-.01	0.12	-.045	0.11	-.01	0.11	-.01	0.11
Percent Hawaiian	-.55*	0.29	-.54*	0.29	-.54*	0.28	-.54*	0.29	-.54*	0.29
Teacher average years of experience	.08	0.22	.08	0.22	.08	0.22	.08	0.22	.08	0.22
Percent teachers with Master's degree (school)	.15***	0.04	.15***	0.04	.15***	0.04	.15***	0.04	.15***	0.04
Log general per pupil fund (district)	.54***	0.12	.53***	0.13	.53***	0.13	.54***	0.13	.54***	0.13
Log school district property tax	-3.3	5.41	3.33	5.42	2.8	5.4	3.42	5.47	3.64	5.5
Rural dummy	-.64	1.28	-0.58	1.37	-.0.71	1.37	-0.6	1.37	-0.5	1.27
Total students (school)	.001***	0.003	.01*	0.003	.01***	0.003	.01***	0.003	.01***	0.003
Adjusted R-squared	0.6		0.6		0.6		0.6		0.6	
Observations	6,256		6,256		6,256		6,256		6,256	

Note: Significance: ***p < 0.01, **p < 0.05, and *p < 0.1. All models include district, county, and year fixed effects.
