# Supplementary material for: Is all greenspace created equal? Assessing the relationship of public parks and greenness on academic achievement in Washington state
Source: PLoS One. 2026 May 6;21(5):e0347301. doi: 10.1371/journal.pone.0347301 (PMC13148709; doi:10.1371/journal.pone.0347301)
Supplement: Attachment — Submitted filename: Response_to_Reviewers_PONE-D-24-39146.docx [file pone.0347301.s004.docx]

**Response to Reviewers for PONE-D-24-39146**

We thank both reviewers and the editor for their comments, which were fundamental in helping us improve the quality of our work. Changes to the manuscript, which has been revised and rewritten following recommendations from the reviewers, are explained in detail below. Text that has been added or significantly modified from the first submission is identified with track changes in the updated document.

**Response to Reviewer 1:**

***Comment 1:*** *The content of the manuscript requires some rearranging in line with the typical layout of a scientific manuscript. In particular the study results should not be described in under ‘I Introduction’ (as they are currently described on page 4). Much of the latter part of the ‘Introduction’ from the last paragraph on page 3 onwards belongs in the Discussion section towards the end of the manuscript.* *I have now read that “PLOS ONE waives all formatting requirements until your manuscript has received a provisional Editorial Accept decision.” I still consider it more standard to have an Introduction that describes background, rationale and aims for the study; then a Methods section; followed by a Results section, separate from a Discussion section. Results should not be described in the Introduction.*

We have moved the study results and the latter part of the “Introduction” section to the newly created “Discussion” section. We have also renamed and rearranged the various sections on the manuscript to reflect the PLOS ONE formatting requirements.

***Comment 2:*** *The benefits of parks for children and adolescents are described throughout the manuscript. At some point the role of parks as a setting for being physically active should be described. Whilst the current study does not examine physical activity (PA) it is important to acknowledge that some beneficial associations between parks and academic performance may have pathways via PA. Positive associations have been shown between PA and academic performance, although not all findings are consistent (e.g. see Marques et al., 2017).*

You are correct that physical activity can be an important pathway via which positive associations can result between parks and academic performance. We have updated the manuscript to acknowledge this role of physical activity and also cited Marques et al. 2017.

Starting at line 44

“Research has found that children and adolescents who live in close proximity to parks are more likely to be physically active [1, 2] have lower obesity rates [3], reduced stress levels, and better emotional wellbeing [4, 5]. Simply being exposed to greenspace has been shown to improve cognitive functioning, memory, and attention spans [5].”

***Comment 3:*** *Please include “, USA” after “western Washington state”.*

We have made the change to the Abstract and the Title to include “Washington state, USA”.

***Comment 4:*** *It would be good to include some studies from beyond the USA. For example, significant associations have been found between surrounding greenery and performance in national standardized school tests in Australia.*

We have added in some additional studies from outside the USA, including the ones mentioned by the reviewer in lines 65-70 as shown below:

“These findings also hold internationally, where researchers in Australia examined the relationship of school vegetation on academic performance for primary school children (grades 3 and 5) and found statistically significant, positive associations with standardized test scores in reading and mathematics [13, 14]. In the United Kingdom, researchers found that 11-year-olds living in greener urban neighborhoods had better spatial working memory [15], which is a strong correlate of academic performance [16].”

and in lines 82-84

“Internationally, a study of German adolescents found mixed results and concluded that there was not an association of increased greenspace and improved academic performance [19].”

***Comment 5:*** *II Data and Methods, Page 5, second paragraph. Please include references for county and city parks and recreation department websites.*

These references have been added.

***Comment 6:*** *“We also assess the overall greenness of the area surrounding the school at various buffer distances…” Please describe the buffer distances.*

We have included the specific buffer distances here in lines 153-155:

“We also assess the overall greenness of the area surrounding the school at various buffer distances (250, 400, and 800 meters) based on the normalized difference vegetation index

(NDVI),”

***Comments 7:*** *Page 6, paragraph 2, “We also include a number of student and school controls in our analysis including race, gender, income, and teacher quality variables”.*

*Please replace “controls” with “covariates”.*

We have changed the term “controls” to “covariates” throughout the manuscript.

***Comments 8:*** *How was “low-income” defined?*

We use OSPI Free and Reduced-Price Meal (FRPM) eligibility as a proxy to define low-income in this study. The source of this data is the information submitted by school districts through the Comprehensive Education Data and Research System (CEDARS). (<https://reportcard.ospi.k12.wa.us/References/Index#FAQ>).

We have included this information in the manuscript in lines 134-136

“At least 40 percent of the students in each grade were identified as being low-income, which is based on their eligibility for free and reduced-price meals at school.”

***Comment 9:*** *Please be consistent when describing the buffer radius, i.e. either 800 m or half a mile; please do not use them interchangeably.*

We have updated the buffer radius to be defined in terms of meters throughout the manuscript.

***Comment 10:*** *Table 1 (should be in Results section). Include values for NDVI, e.g. mean and standard deviation or median and range.*

As per the reviewer’s suggestion, we have added the standard deviations of the variables of interest at all three school levels. We have also created a separate Table 2 in “Parks and Greenspace Data” subsection within the “Materials and Methods”, which includes the mean and standard deviation values for NDVI for each buffer distance for both Fall and Spring. We decided to keep these tables in the Materials and methods section since we first reference the variables and summary statistics in this section.

***Comment 11*:** *Table 2 (should be in Results section)*

Table 2 has been renamed Table 3. We have moved it to the “Results” section and updated it to include the standard errors.

***Comment 12:*** *Rather than “(dummy)” after “Park 800m”, it would be clearer to write “Park within 800m”.*

We have updated all the tables to reflect this change.

***Comment 13:*** *Last sentence of section III appears to be missing a final word: “If parks are more likely to have greater tree cover, then these results coincide with our findings that tree covered parks have a stronger relationship with”.*

Thank you for bringing this to our attention, we have updated the sentence.

Line 343: “If parks are more likely to have greater tree cover, then these results coincide with our findings.”

**Response to Reviewer 2:**

***Comment 1:*** *I indicated that the statistical analysis was not performed rigorously because it appears that the authors want to make a causal inference, and I don't think that the methods support this. The methods may be rigorous enough if they are wanting to make different conclusions, and perhaps added additional control variables. More details below:
This paper examines the relation for greenspace around schools to academic achievement at the school level. The paper utilizes multiple metrics of greenspace to determine that schools that have a public park located within ½ mile radius have higher student achievement levels than do schools without a park in the ½ mile radius. This relation was found only among middle schools but not among elementary or high schools. Greenspace as measured by the NDVI was not associated with student achievement.*

*Overall, I found the paper to be interesting but felt that the description of the findings and conclusions were not completely in line with the analysis methodology used.
The authors use causal language describing ‘impact’ of greenspace throughout the paper. Based on my understanding of their methods, I don’t think that any causal conclusions can be drawn.*

You are correct that the intention of the manuscript is not to establish a causal relationship between greenspace and academic performance. Therefore, we have updated the title “Is all greenspace created equal? Assessing the *relationship* of public parks and greenness on academic achievement in Washington state, USA” and the language throughout the manuscript to clarify that our study attempts to establish a correlational, not causal, relationship between the two.

***Comment 2:*** *The inclusion of multiple measures of greenness and various control variables is a strength of the study as the authors discuss, but I wonder why other school neighborhood variables were not included. It seems that including these would be necessary to advancing the argument that parks, rather than neighborhood resources more generally, are related to achievement.*

We agree that including other neighborhood variables would have helped us better identify the relationship between parks and academic performance. Unfortunately, we did not have access to good data on school neighborhood characteristics as the school attendance areas do not overlap directly with areas represented in other census data (e.g. census blocks or zip codes). While this is a limitation of the study, some variables on income and student demographics could capture neighborhood characteristics around the schools, more so for elementary than middle or high schools, which have larger catchment areas.

We briefly address this in the Discussion section as a partial reason for why the elementary school results are not statistically significant in lines 354-362:

“One potential factor explaining why the results are positive and significant only at the middle school level is that middle schoolers are able to be more independent and thus, could be more likely to spend time in parks in close proximity to their school whereas elementary school students are more likely to spend time in parks that are near their residence since they still need parental supervision. We hypothesize that interactions with public parks also have benefits to elementary age children’s academic performance, but a more appropriate study would focus on park proximity at the neighborhood level rather than the school.”

***Comment 3:*** *Associations between school racial/ethnic composition and school achievement are presented without discussion of systemic racism as a reason for these differences.*

In the results section, we have added some citations to support that our coefficients regarding race and SES (measured by the low-income variable) are consistent with previous literature. However, since this study is focused on parks and greenspace, not race and income, we have refrained from adding too much discussion on the potential drivers of these results as it is outside the scope of our paper.

See lines 230-240:

“Additional results show that the percent of low-income students, as a proxy for measuring household income, has a significant and negative relationship with educational outcomes across all three school levels. This suggests that grades with a higher percentage of low-income students experience a significant reduction in the percentage of those who meet or exceed proficiency standards on assessment tests, which is consistent with previous literature examining the relationship between academic achievement and socioeconomic status [33]. Racial and gender compositions vary in their significance and magnitude across school levels. Generally speaking, the percentage of Asians tends to be associated with an increasing percentage of students that met or exceeded test standards, while the percentages of Black, Hispanics, and Native Americans, generally have a negative association across all school levels, which is also consistent with previous literature [34].”

***Comment 4:*** *Analyses were done predicting school level aggregates of achievement, but findings are discussed in terms of how parks might impact individual children’s behavior. Is this a correct interpretation of results? Have the authors considered whether this may be an example of the ecological fallacy?*

In our study, we focus on average outcomes for grade levels, but recognize that individual students will have a range of experiences and engagement with parks. Since we do not have data on student attendance with parks, our study only highlights the correlational outcomes and associations between parks and academic performance at the school grade level. Nonetheless, we understand that our aggregate level analyses and individual level discussion can create confusion for the readers. Thus, as per the reviewer’s comment we have changed the language to focus more towards school level outcomes rather than individual level outcomes and on making it less causal and more correlational.

***Comment 5:*** *Additionally, I was surprised that the authors described the results of their study in the introduction. This may be a disciplinary difference.*

We have taken out the summary of results from the Introduction section, which is a standard convention in economic journals.

**Response to the Academic Editor:**

***Comment 1:*** *Please ensure that your manuscript meets PLOS ONE's style requirements, including those for file naming. The PLOS ONE style templates can be found at*

[*https://journals.plos.org/plosone/s/file?id=wjVg/PLOSOne_formatting_sample_main_body.pdf*](https://journals.plos.org/plosone/s/file?id=wjVg/PLOSOne_formatting_sample_main_body.pdf)

*and*

[*https://journals.plos.org/plosone/s/file?id=ba62/PLOSOne_formatting_sample_title_authors_affiliations.pdf*](https://journals.plos.org/plosone/s/file?id=ba62/PLOSOne_formatting_sample_title_authors_affiliations.pdf)

We have updated the manuscript to reflect PLOS ONE’s style requirements.

***Comment 2:*** *In your Methods section, please include additional information about your dataset and ensure that you have included a statement specifying whether the collection and analysis method complied with the terms and conditions for the source of the data.*

We have added in the manuscript that the data is available for public use (per the OSPI website: <https://ospi.k12.wa.us/data-reporting/data-portal>) and have included an in-text citation with a link to access the data (reference [24]).

***Comment 3:*** *When completing the data availability statement of the submission form, you indicated that you will make your data available on acceptance. We strongly recommend all authors decide on a data sharing plan before acceptance, as the process can be lengthy and hold up publication timelines. Please note that, though access restrictions are acceptable now, your entire data will need to be made freely accessible if your manuscript is accepted for publication. This policy applies to all data except where public deposition would breach compliance with the protocol approved by your research ethics board. If you are unable to adhere to our open data policy, please kindly revise your statement to explain your reasoning and we will seek the editor's input on an exemption. Please be assured that, once you have provided your new statement, the assessment of your exemption will not hold up the peer review process.*

The data availability statement has been included with the submission form. The data used in the study is publicly available and available for sharing.

***Comment 4:*** *We note that Figure 1 in your submission contain map/satellite images which may be copyrighted. All PLOS content is published under the Creative Commons Attribution License (CC BY 4.0), which means that the manuscript, images, and Supporting Information files will be freely available online, and any third party is permitted to access, download, copy, distribute, and use these materials in any way, even commercially, with proper attribution. For these reasons, we cannot publish previously copyrighted maps or satellite images created using proprietary data, such as Google software (Google Maps, Street View, and Earth). For more information, see our copyright guidelines:* [*http://journals.plos.org/plosone/s/licenses-and-copyright*](http://journals.plos.org/plosone/s/licenses-and-copyright)*. We require you to either (a) present written permission from the copyright holder to publish these figures specifically under the CC BY 4.0 license, or (b) remove the figures from your submission.*

The map in Figure 1 uses Open Street Map (OSM) images in QGIS. OSM is a publicly available, open source resource, per their copy right and licensing agreement seen here: <https://www.openstreetmap.org/copyright>

We have added an attribution to OSM under Figure 1 as directed by their attribution guidelines.
