# Supplementary material for: Is all greenspace created equal? Assessing the relationship of public parks and greenness on academic achievement in Washington state
Source: PLoS One. 2026 May 6;21(5):e0347301. doi: 10.1371/journal.pone.0347301 (PMC13148709; doi:10.1371/journal.pone.0347301)
Supplement: Attachment — Submitted filename: RR3 Response to Reviewer Comments.docx [file pone.0347301.s006.docx]

PONE-D-24-39146R2

Is all greenspace created equal? Assessing the relationship of public parks and greenness on academic achievement in Washington, USA

PLOS One

**Response to Reviewer #3’s Comments**

Reviewer #3: The authors have effectively addressed reviewer responses. I did note a couple of issues that should be corrected before publication and I raise one substantive issue that I ask the authors to consider:

Pg 6, referring to line 116 - there is a comment in the margins that says "While the study area includes only urban counties, our sample includes both rural and urban school districts from the National Center for Educational Statistics (NCES) [23]. Thus, our findings pertain to access to park in both rural and urban areas." That seems to be a more clear way of articulating this point and responding to the reviewer's concern, with perhaps a simple edit - "While the study area includes only urban counties, there are both urban and rural school districts (by definition of ...(NCES) in these counties. Thus, our findings pertain to access to parks in both rural and urban areas.

We thought we had deleted this comment, which was from a previous revision that we already addressed. However, we have now moved this information to the School Data section [line 125-127] since it describes specific school characteristics, which we think is a better fit and included the sentence:

“To account for schools located in more rural areas in western Washington, we include data from the National Center for Educational Statistics (NCES) that classifies schools as rural based on their distance from an urban area [23]. ”

Line 146 Table 1 - this isn't a big deal, but it would improve clarity and avoid confusion if the table were divided into two sections (upper for data that are numbers with a relevant column heading - not means and SDs, and lower with the means and SDs heading for data relevant to representing means and SDs. )

We have rearranged the table according to your suggestions, which we agree, makes more sense.

Line 224 - "within a half mile" is a different unit of measurement than 800 meters, but it is a helpful one for audiences that think in miles not km. Perhaps just parenthetically noting the equivalence, like "...having a public park within a half mile radius (800 meters) of a school...."

We have made this change in the manuscript (line 226).

Line 316 - I believe the authors missed changing this to 4.2

Thank you, we have made this change (line 311).

Line 373 - 376 - by saying "the latter" it seems you are saying that your results represented a negative relationship as opposed to no relationship. The use of the dichotomous former/latter way of speaking doesn't work when you want to refer to something that is the middle and third option.

We have changed the language here to take out the word “latter” (line 434).

Limitations section - A commonly discussed limitation of the use of NDVI in buffers is that it represents data "as the crow flies" and does not account for potential lack of access to that green space by, for example a highway, fencing, etc. Driving/walking routes to that greenspace are often used to address this. I'm not suggesting that data be re-analyzed, but it would be helpful to note in the Limitations section that NDVI "as the crow flies" does have its drawbacks.

We have added a line [388-390] to account for this limitation:

“One limitation of using buffers for the park and NDVI data is that it represents distance “as the crow flies” and does not account for potential barriers to access (e.g. highways, fences, etc.).”

Discussion- I understand that NDVI was a control for this study, not the focus of the analysis with outcome data, and that NDVI data was not disparate from that documented in past studies. But, it would help to note in the discussion that your finding of no NDVI/outcome relationship is in contrast to some previous findings, even those studies with similar amount of greenness, but overall is consistent with the mixed results in the literature.

The variation in NDVI in our study is consistent with other studies, specifically Kuo et al. (2021) and Browning, et al. (2018), that also found no statistically significant relationship between NDVI and academic performance in for former, and a small negative relationship in the latter. We briefly mention this in the Discussion section, but we have more discussion on it in the Limitations section, as stated below in starting in line 406.

“we do note that the mean and standard deviation of our NDVI values are in line with other studies that have examined the relationship between NDVI and academic performance and have similarly, found no statistically significant relationship [12] or a near zero, but negative and statistically significant relationship [18].”

This is my substantive suggestion for consideration:
Bottom of pg 12 - there is increasing interest in testing the equigenic effect - when children from lower SES backgrounds (as measures by family, school or community economic data) benefit RELATIVELY MORE from nature than children from higher SES backgrounds. It seems that lines 240 - 242 only tell part of the important story. Is it possible to analyze the middle school data stratified by a relevant SES variable, rather than simply controlling for it?

We analyzed the data stratified by the percent of low income students in middle schools, looking at the bottom poorest 25% of schools (60% or more of the students are reported as low-income) and the wealthiest 25% of schools (less than 20% of students are reported as low-income). The coefficient for the low-income schools is negative and the coefficient for the wealthiest schools is positive, though neither are statistically significant. For the middle 50% of schools, the coefficient was significant and similar in magnitude to the main results from Table 3 in the paper. We have compiled the results in the table below (but we have not added this table to the paper).

**Stratified Sample on Percent Low-income Students for Middle Schools**

| **Outcome variable: Percent met standard** | **Bottom 25% (Richest)** | | **Middle 50%** | | **Top 25% (Poorest)** | |
| --- | --- | --- | --- | --- | --- | --- |
|  | **Coef.** | **SE** | **Coef.** | **SE** | **Coef.** | **SE** |
| Park within 800 m | 4.53 | 2.75 | 4.4*** | 1.6 | -0.59 | 2.17 |
| NDVI (800 m spring buffer) | 25.55** | 11.68 | 1.5 | 12 | 5.66 | 25.12 |
| District, county, and time fixed effects | Y | | Y | | Y | |
| Adjusted R-squared | 0.6 | | 0.33 | | 0.4 | |
| Observations | 1,511 | | 3,297 | | 1,417 | |

Ultimately, we feel that these results are too inconclusive to report and are outside the scope of our current paper, though we think that this is an important question worth exploring further in a more robust way and would like to do so in the future. Ideally we would like to have more data on park quality and potentially other neighborhood characteristics as additional control variables to have a more robust model for understanding how the relationship between parks and academic performance might vary by SES. For example, it may be the case that parks near lower income schools are of poorer quality and/or may be more susceptible to crime or homelessness, which could be driving these preliminary results.
